# Supplementary material for: The Construction and Exploration of a Comprehensive MicroRNA Centered Regulatory Network in Foxtail Millet (Setaria italica L.)
Source: Front Plant Sci. 2022 May 6;13:848474. doi: 10.3389/fpls.2022.848474 (PMC9121102; doi:10.3389/fpls.2022.848474)
Supplement: Supplementary file 1 [file Data_Sheet_1.zip › Sit-miR2118e_Seita.3G360700.1_30_TPlot.pdf]

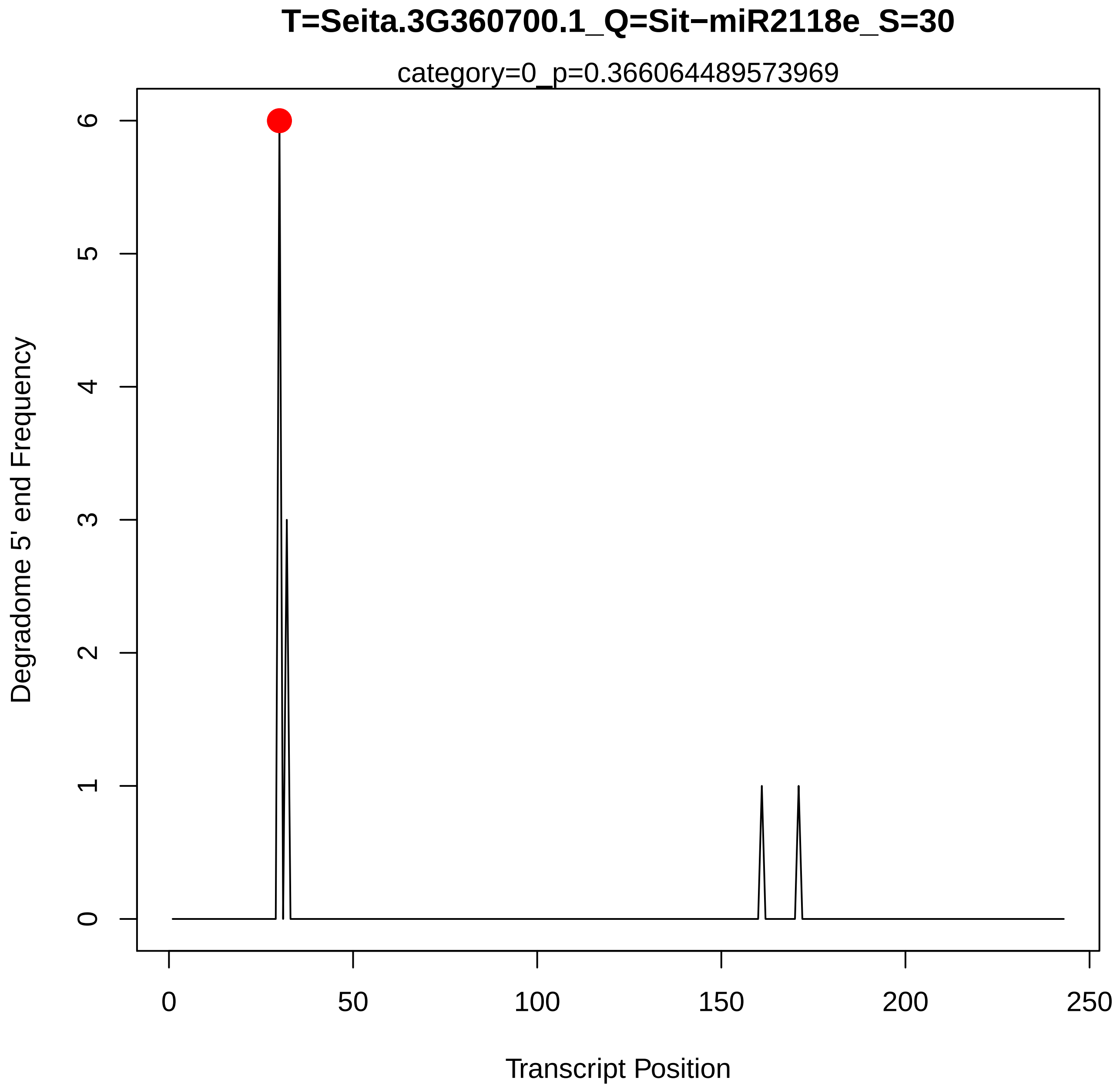
T=Seita.3G360700.1_Q=Sit−miR2118e_S=30
category=0_p=0.366064489573969
Degradome 5' end Frequency
Transcript Position
0
1
2
3
4
5
6
0
50
100
150
200
250
